# Locoregional Control from Complete Peritoneal Cytoreduction in Patients with Extraperitoneal Colorectal Cancer Metastases: An Entropy Balanced Analysis

**DOI:** 10.1245/s10434-026-19750-7

**Published:** 2026-05-11

**Authors:** M. Daniyal Tanweer, Isaac B. Paz, Thinzar Lwin, Laleh Melstrom, Yuman Fong, Gagandeep Brar, Dani Castillo, Pashtoon Kasi, Marwan Fakih, Mustafa Raoof

**Affiliations:** 1 Department of Surgery, City of Hope National Medical Center, Duarte, CA USA; 2 Department of Medical Oncology and Therapeutics Research, City of Hope National Medical Center, Duarte, CA USA

**Keywords:** Colorectal cancer, Peritoneal metastases, Extraperitoneal metastases, Cytoreductive surgery, Overall survival, Progression-free survival

## Abstract

**Purpose:**

We evaluated the role of complete cytoreductive surgery (CRS) for locoregional disease control in patients with colorectal cancer with peritoneal metastases (CRC-PM) and concurrent extraperitoneal metastases (EPM).

**Methods:**

Institutional data identified patients with CC-0/1 CRS for CRC-PM. Patients with peritoneal disease only (PDO) were compared with those with concomitant EPM. Co-primary outcomes were progression-free survival (PFS) and peritoneal progression-free survival (p-PFS). The secondary outcome was overall survival (OS). Covariate imbalance was addressed using entropy balancing. Survival was analyzed using a weighted Cox proportional hazards model. Subgroup analyses were performed to identify favorable prognostic groups.

**Results:**

In total, 83 patients were included: 31 (37.4%) had EPM. Baseline characteristics were comparable. Within the EPM cohort, liver-only metastases were most common (58%), followed by lung-only metastases (26%). EPM were managed at index CRS in 61%, before CRS in 19%, and deferred or untreated in 19% of patients. Unadjusted analyses demonstrated comparable median OS (PDO vs. EPM: 39 vs. 34 months, *p *= 0.551) and median p-PFS (PDO vs. EPM: 17 vs. 15 months, *p *= 0.346) but longer median PFS in the PDO group (14 vs. 5 months, p<0.001). After entropy balancing, patients with EPM had worse OS (31.6 vs. 38.7 months; hazard ratio [HR] 2.12; 95% confidence interval [CI] 1.02–4.37; *p *< 0.05) and PFS (5.0 vs.12.8 months; HR 2.7; 95% CI 1.58–4.5; *p *< 0.001) but similar p-PFS (13.4 vs.14.9 months; HR 1.10; 95% CI 0.58–1.77; *p *= 0.622). Subgroup analyses demonstrated comparable OS and p-PFS for patients with PDO and those with single-site liver or lung EPM.

**Conclusion:**

Complete CRS provides durable locoregional disease control in patients with EPM.

Colorectal cancer (CRC) remains one of the leading causes of cancer-associated morbidity and mortality.^1^ The peritoneal cavity is the third most common site of metastasis for CRC after liver and lungs.^2^ Colorectal peritoneal metastases (CRC-PM) represent a unique clinical entity with worse survival compared to liver- or lung-only metastases: median survival is less than 16 months despite modern chemotherapy.^3^

Complete cytoreductive surgery (CRS) – cytoreduction score (CC) 0/1 – is an established treatment for patients with peritoneal disease only (PDO), offering the possibility of durable disease control and 5-year survival rates approaching 35%.^4–6^ Though the concomitant use of hyperthermic intraperitoneal chemotherapy (HIPEC) for CRC-PM has been called into question, complete CRS remains the strongest predictor of improved survival.^7^ However, these superior outcomes are contingent on careful patient selection at expert centers and meticulous multidisciplinary and perioperative care.^8^

Approximately 86% of patients with CRC-PM have non-isolated peritoneal metastases (i.e., concurrent extraperitoneal metastases [EPM] involving lung, liver and/or retroperitoneal lymph nodes).^9^ Historically, this subset had been excluded from studies investigating the utility of CRS ± HIPEC in disease control for CRC-PM,^10^ largely because of the prevailing understanding that EPM reflects disseminated systemic disease and therefore might be incompatible with the goals of complete cytoreduction.^11^

Recent advances in systemic therapy^12^, improved surgical selection, and a growing understanding of the heterogeneity of EPM with CRC-PM^13,14^ have expanded surgical indications. CRS is usually pursued to address the life-limiting disease in the peritoneum that frequently leads to decreased quality of life and treatment discontinuation. However, it remains unknown whether the durability of locoregional control after CRS in patients with EPM is comparable to that in those with isolated PDO.

A few studies have established the feasibility of a combined resection for CRC-PM and liver metastasis with favorable overall survival (OS).^15,16^ Similarly, patients with CRC-PM and pulmonary metastases have been shown to have OS comparable to that in those with PDO.^13^ Although these studies have shown considerable improvements in CRS outcomes for patients with EPM over the last two decades, recurrence rates (65–75%) after complete resection remain high.^17^

In this contemporary comparative analysis from a National Cancer Institute-designated comprehensive cancer center, we evaluated the oncological outcomes in patients with CRC-PM undergoing curative-intent CRS. We compared patients with peritoneal disease and EPM (EPM group) and those with PDO. Our primary objective was to determine whether the presence of EPM was associated with inferior locoregional disease control, measured as peritoneal progression-free survival (p-PFS) or overall disease control measured as progression-free survival (PFS). Our secondary objective was to compare OS in both groups. In exploratory analysis, we sought to identify favorable subsets of patients with EPM that may inform patient selection for CRS.

## Methods

### Database and Patients

This was a single-institution retrospective analysis of a prospectively maintained database of consecutive patients with CRC-PM undergoing CRS ± HIPEC, either with or without EPM, from 2009 to 2024. Patient characteristics, treatment histories, and follow-up data were abstracted from the hospital records within the institutional electronic medical record system. The study was approved by the institutional review board.

### Interventions and Procedures

CRS candidacy was determined after a multidisciplinary discussion. Generally, CRS was considered for patients if they had (1) adequate functional status enabling postoperative recovery; (2) response or stability of disease on preoperative chemotherapy; (3) a high probability of complete resection for all visible metastases on pre-CRS laparoscopy; and (4) an acceptable risk of postoperative complications and ensuing quality of life. CRS consisted of peritonectomy procedures and visceral resections, performed either via a midline laparotomy or using minimally invasive techniques in selected cases.^18^ Disease burden was evaluated using Sugarbaker’s Peritoneal Carcinomatosis Index,^19^ and completeness of cytoreduction was reported using the CC score.^20^ After the release of the PRODIGE 7 trial results in 2021, institutional practice shifted away from the routine use of HIPEC for CRC-PM. When HIPEC was used, it was administered at an average temperature of 42 °C with mitomycin C (25 mg/m^2^) for 90 minutes.

### Follow-up

Postoperatively, patients received a baseline computed tomography (CT) scan and tumor markers at 6–8 weeks. Chemotherapy was classified as adjuvant, maintenance, or palliative. Adjuvant therapy was defined as chemotherapy given with a pre-defined duration of therapy (typically 2–6 months) in the absence of radiographic disease. Maintenance therapy was defined as less intense chemotherapy (typically 5-fluorouracil/leucovorin ± bevacizumab) continued indefinitely in the absence of radiographic disease. Palliative chemotherapy was defined as chemotherapy for radiographic residual metastatic disease. Patients were followed every 2–3 months for the first 3 years and every 6 months thereafter with routine physical examination, assessment of serum tumor markers (carcinoembryonic antigen) and thoracoabdominal and pelvic CT scans or magnetic resonance imaging. The first detection of peritoneal or new EPM disease after index CRS was reported as disease progression. Progression of existing unresected EPM (without new EPM) lesions was also considered progression of disease. Patients with elevated tumor markers without contrast CT appearance of disease were further evaluated with positron emission tomography-CT and/or magnetic resonance imaging.

### Survival Outcomes

Primary outcome was PFS, defined as the time from day of index CRS to peritoneal/extraperitoneal (or both) progression of disease or death. Co-primary outcome was p-PFS, defined as the time from index CRS to peritoneal progression. The secondary outcome was OS, which was defined as the time from date of CRS to last follow-up and/or death.

### Statistical Analyses

Categorical variables were reported as frequencies and percentages of the entire cohort, and continuous variables were represented as medians and interquartile ranges (IQRs). T test or the Mann–Whitney U test was used for continuous variables. Pearson’s χ^2^ test or Fisher’s exact test was used to compare categorical variables. For the unadjusted survival analysis, the Kaplan–Meier method was used, and survival was compared using the log rank test.

Entropy balancing was performed to address covariate imbalance.^21,22^ We balanced the groups on known prognostic factors for patients with CRC-PM. These included age, sex, previous debulking surgery, Peritoneal Carcinomatosis Index, CC score, primary tumor location, and receipt of preoperative chemotherapy. Entropy balancing effectively balances cases with controls and, given the distribution of our data, it was considered superior to alternative covariate adjustment methods such as propensity score matching.^23^ Using assigned weights, we used cox regression models for OS, PFS and p-PFS to plot adjusted survival curves. The proportional hazards assumption was formally assessed using Schoenfeld residuals; covariates demonstrating non-proportionality were addressed through stratified Cox regression, and the exposure of interest (i.e., EPM) satisfied the proportional hazards assumption. Adjusted hazard ratios (HRs) with 95% confidence intervals (CIs) for the three primary outcomes were reported. Adjusted median OS, PFS, and p-PFS, complementary to the relative effect estimates derived from the Cox model, were estimated using a flexible parametric survival model.

Exploratory subgroup analysis, for site-specific (PDO vs. peritoneal disease [PD] + lung vs PD + liver) endpoints were reported for the unbalanced population. A p-value <0.05 was considered statistically significant. Analysis was performed using STATA (v 17.0).

## Results

Patient characteristics are summarized in Table 1. In total, 83 patients underwent complete CRS, of whom 52 (62.6%) had PDO. Baseline demographics and clinicopathological factors were comparable between the two study groups. Within the EPM cohort, liver-only metastases were most common (58%), followed by lung-only metastases (26%), and nodal and multisite involvement was observed less frequently. Patients with liver-only metastases had a median of two lesions (range 1–6), whereas those with lung-only metastases had a median of one lesion (range 1–4).

**Table 1:** Baseline characteristics

Variable	Total (N=83)	PDO (N=52)	EPM (N=31)	p-Value
Age	56 (48.6–66)	56.1 (48.8–66.7)	55.1 (48.1–64)	0.55^a^
Sex				0.35^b^
Female	43 (52)	29 (56)	14 (45)	
Male	40 (48)	23 (44)	17 (55)	
Race/ethnicity				0.19^b^
White	41 (49)	27 (52)	14 (45)	
Asian	22 (27)	16 (31)	6 (19)	
Latino	11 (13)	6 (12)	5 (16)	
Other	9 (11)	3 ( 6)	6 (19)	
Charlson Comorbidity Index score	7 (6–9)	7 (6.5–8)	8 (6–9)	0.61^a^
Primary tumor location				0.21^b^
Right/transverse	34 (41)	24 (46)	10 (32)	
Left/sigmoid	41 (49)	25 (48)	16 (52)	
Rectosigmoid/rectum	8 (10)	3 (6)	5 (16)	
Primary tumor differentiation				0.71^b^
Well	17 (20)	11 (21)	6 (19)	
Moderate	52 (63)	31 (60)	21 (68)	
Poor	14 (17)	10 (19)	4 (13)	
KRAS status				0.47^b^
Wild-type	37 (45)	21 (40)	16 (52)	
Mutated	18 (22)	11 (21)	7 (23)	
Unknown	28 (34)	20 (38)	8 (26)	
Previous debulking surgery	14 (17)	11 (21)	3 (10)	0.23^b^
PCI category				0.83^b^
≤10	51 (61)	33 (63)	18 (58)	
11–15	16 (19)	10 (19)	6 (19)	
>15	16 (19)	9 (17)	7 (23)	34
CCR				0.21^b^
0	72 (87)	47 (90)	25 (81)	
1	11 (13)	5 (10)	6 (19)	
HIPEC at index CRS	45 (54)	32 (62)	13 (42)	0.083^b^
Preoperative chemotherapy	66 (80)	38 (73)	28 (90)	0.091^b^
Postoperative chemotherapy				0.99^b^
Adjuvant only	22 (27)	14 (27)	8 (26)	
Maintenance (± adjuvant)	11 (13)	7 (13)	4 (13)	
Palliative	50 (60)	31 (60)	19 (61)	

Data are presented as n (%) for categorical variables and median (interquartile range) for continuous variables

^a^Student’s T test, Mann–Whitney U test

^b^ Pearson’s χ^2^ test or Fisher’s exact test

CCR, Completeness of Cytoreduction; CRS, cytoreductive surgery; EPM, co-existing extraperitoneal metastases; HIPEC, hyperthermic intraperitoneal chemotherapy; PCI, Peritoneal Carcinomatosis Index; PDO, peritoneal disease only

### EPM Management

**Fig. 1:**
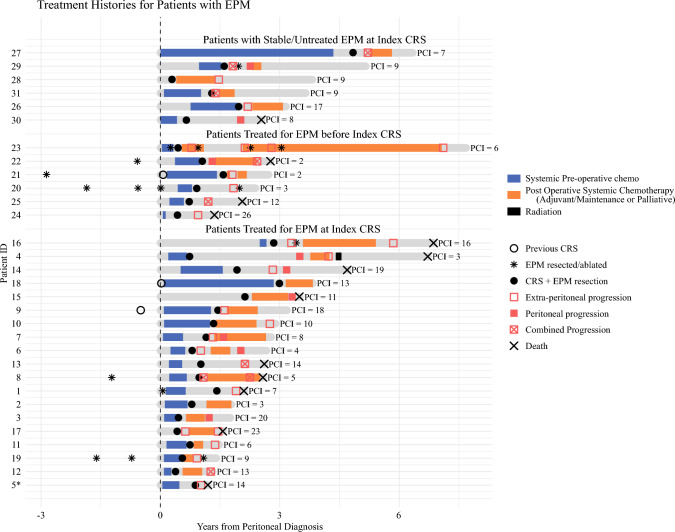
Longitudinal treatment histories of patients in the extraperitoneal metastases (EPM) cohort, stratified by timing of EPM treatment. *Patient received palliative chemotherapy. CRS, cytoreductive surgery; PCI, Peritoneal Carcinomatosis Index at CRS. Combined progression: peritoneal + extraperitoneal progression

Most patients underwent treatment of extraperitoneal disease at the time of index CRS (61%), whereas 19% received treatment before CRS, and a similar number had untreated EPM at the time of surgery. Among the patients treated for EPM at the time of CRS, three had previous EPM resections/ablations. Of the six patients treated for EPM before CRS, four had undergone at least one resection/ablation treatment. All patients with stable/untreated EPM at the time of CRS had lung metastases. Detailed treatment histories are shown in Fig. 1.

### Unadjusted Survival Analysis

**Fig. 2:**
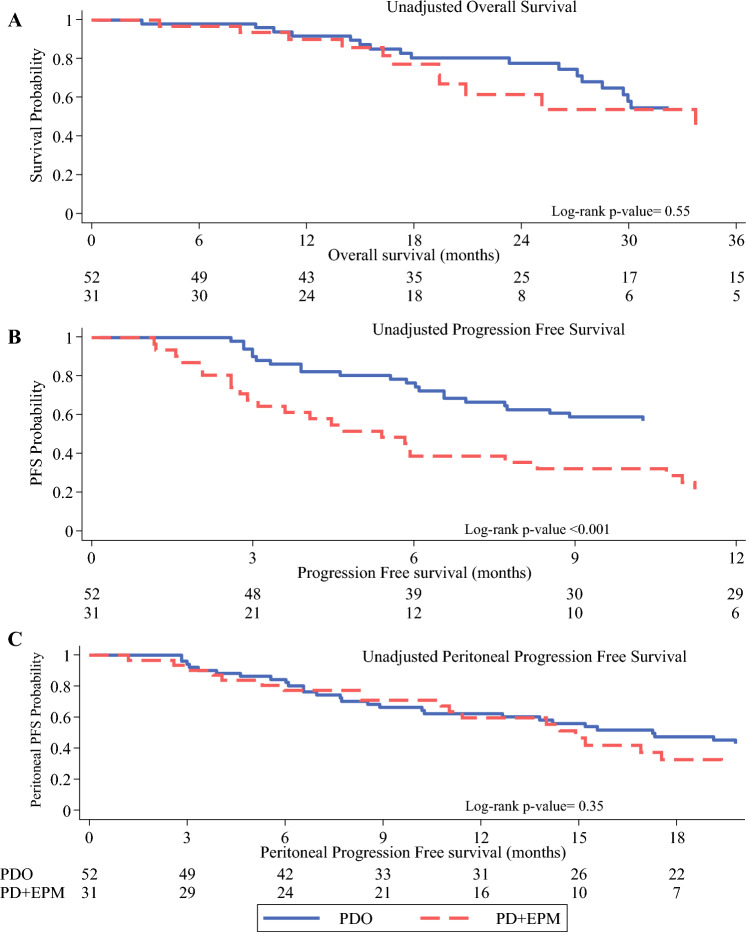
Kaplan–Meier unadjusted survival analysis. **A** Overall survival, **B** progression-free survival (PFS), and **C** peritoneal PFS for patients with colorectal cancer and peritoneal disease (PD) with and without extraperitoneal metastases (EPM)

The median follow-up for the entire cohort was 28.7 months. Median OS was similar between groups: 39.4 months in the PDO cohort and 33.7 months in the EPM cohort (*p *= 0.551). However, median PFS was significantly longer in patients with PDO (13.8 vs. 5.4 months, p < 0.001), and p-PFS was comparable between PDO and EPM (17.3 vs. 14.9 months, *p* = 0.346) (Fig. 2A–C).

### Adjusted Survival Analysis

**Fig. 3:**
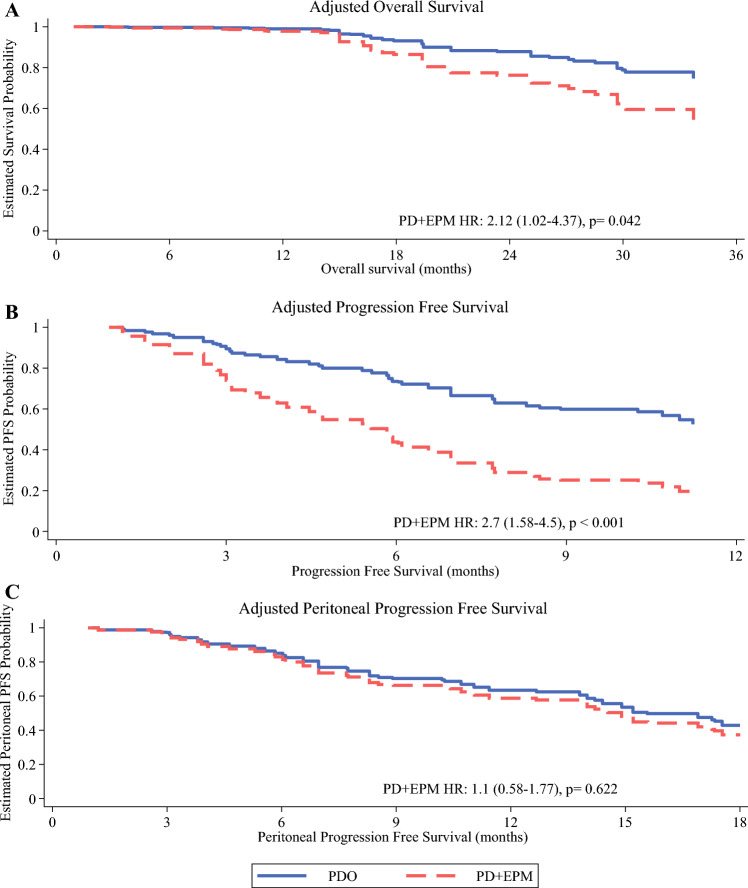
Adjusted survival analysis. Entropy balanced Cox survival curves. Adjusted **A** overall survival, **B** progression-free survival (PFS), and **C** peritoneal PFS for patients with colorectal cancer and peritoneal disease (PD) with and without extraperitoneal metastases (EPM). CI, confidence interval; HR, hazard ratio

In the entropy-balanced analysis, patients with PDO experienced significantly improved OS compared with those with EPM (HR 2.12; 95% CI 1.02–4.37; *p *= 0.042), with an adjusted median OS of 38.7 versus 31.6 months, respectively. EPM was associated with inferior PFS (HR 2.70; 95% CI 1.58–4.50; *p *< 0.001), corresponding to adjusted median PFS of 5.0 months compared with 12.8 months for PDO. In contrast, adjusted p-PFS did not differ between groups (HR 1.10; 95% CI 0.58–1.77; *p *= 0.622), with comparable median durations of 14.9 and 13.4 months for PDO and EPM, respectively (Fig. 3A–C).

### Subgroup Analysis by EPM Site

**Fig. 4:**
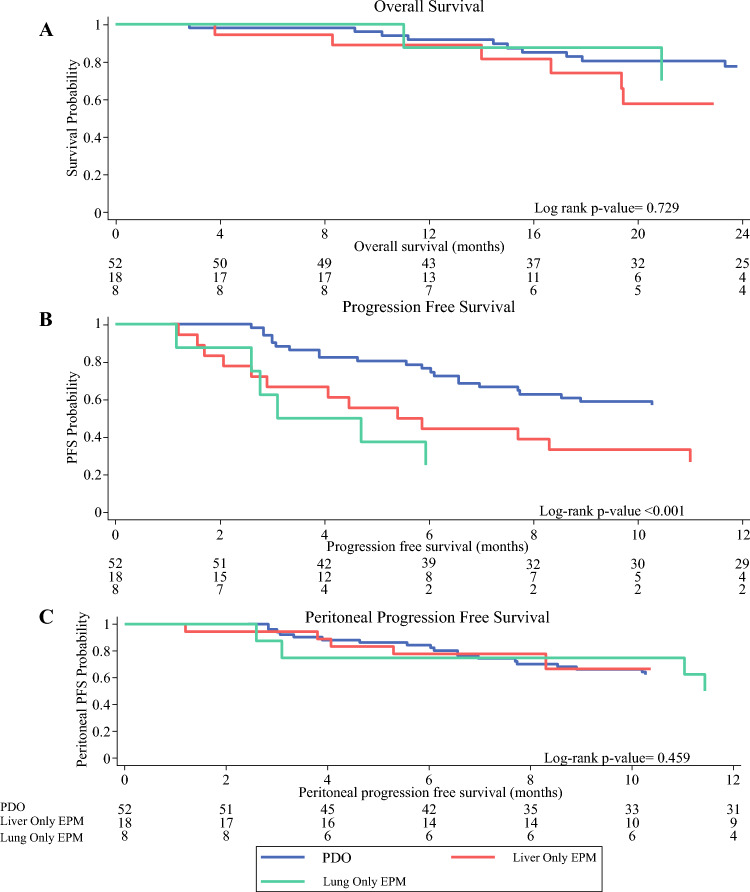
Subgroup analysis stratified by site of extraperitoneal metastases (EPM). **A** Overall survival, **B** progression-free survival (PFS), and **C** peritoneal PFS for patients with colorectal cancer and peritoneal disease stratified by single-site EPM only

In an exploratory analysis evaluating the impact of EPM site, median OS was comparable between patients with PDO and those with liver-only EPM (39.4 vs. 33.7 months), whereas median OS was not reached in patients with lung-only metastases (Fig. 4A). PFS differed by subgroup, with shorter PFS observed in patients with lung-only or liver-only EPM compared with those with PDO (*p* < 0.001) (Fig. 4B). In contrast, p-PFS survival did not differ significantly across the three subgroups (Fig. 4C).

## Discussion

The durability of peritoneal disease control with CRS in patients with EPM has not been previously evaluated. CRC involving multiple sites is seen as a poor prognostic factor. The prevailing understanding is that patients with EPM may have worse peritoneal disease control from CRS than those with PDO, potentially limiting the utility of CRS in such cases. Conversely, the main finding of the present study is that peritoneal disease control is comparable in patients with CRC-PM with or without EPM. Although OS appeared worse among patients with CRC-PM with EPM undergoing CRS, this was largely driven by extra-peritoneal disease progression rather than peritoneal disease progression.

Approximately 50% of patients with CRC develop metastases over the course of their disease,^24^ and 15% have peritoneal disease.^25^ Peritoneal disease governs the survival and quality of life in patients with metastatic disease from CRC limited to the peritoneal cavity.^10^ Studies have classified peritoneal metastases as biologically distinct. Analysis of 1976 tumors from patients with CRC from the COIN trial (oxaliplatin and fluoropyrimidine chemotherapy ± cetuximab) revealed significantly enriched BRAF and microsatellite instability in patients with PDO.^26^ Laoukili et al.^27^ demonstrated that CRCs that develop PDO exhibit an aggressive, mesenchymal phenotype with a high degree of chemoresistance. When coupled with systemic dissemination in the form of EPM,^28^ this biological profile may reasonably portend poorer survival outcomes.

Most (86%) patients with peritoneal disease also have at least limited EPM.^29^ Although the peritoneum is the life-limiting disease location for these patients, historically, peritoneum-directed interventions, such as CRS, have been met with nihilism because of concerns about the limited durability of locoregional control in the setting of EPM.^30^ Multiple studies report discouraging survival outcomes for patients with CRC-PM in the setting of EPM.^31^ For instance Baratti et al.^32^ reported 5-year OS of 16.5% for 27 patients with EPM (liver and lung metastases) versus 52% in patients with PDO. However, 80% of EPM patients showed disease relapse at extraperitoneal sites after CRS/HIPEC. 

Resection of multiorgan metastases in CRC has been evaluated in the ORCHESTRA trial.^33^ In this trial, patients were eligible if at least 80% tumor debulking was deemed feasible by resection, radiotherapy, and/or thermal ablative therapy at the start of first-line palliative systemic therapy. After an initial 3–4 cycles of systemic therapy, patients were randomized to either maximal tumor debulking followed by resumption of systemic therapy in the intervention arm or continued systemic therapy in the control arm. Most (42–45%) of the patients had liver and lung metastases, and about one-third had peritoneal metastases along with EPM. There was no difference in the primary outcome, OS, between the two arms. The median OS was 27.5 months in the standard arm and 30.0 months in the experimental arm (adjusted HR 0.88; 95% CI 0.70–1.10; *p *= 0.225). The median PFS was 10.4 months in the standard arm and 10.5 months in the experimental arm (adjusted HR 0.83; 95% CI 0.67–1.02; *p *= 0.076). In the present study, 31 (37.3%) patients had concomitant EPM, the majority (61%) of which was resected at the time of index CRS. Unlike in the ORCHESTRA trial, where complete debulking/cytoreduction was achieved in only 38% of patients, complete cytoreduction was achieved in all patients in the present study. Although the presence of EPM in our cohort was associated with inferior adjusted median OS compared with PDO (31.6 vs 38.7 months), the adjusted 3-year survival rate of 42% compared favorably with a rate of 55% in patients with PDO. We attribute this improvement to careful patient selection and advancements in systemic therapies. Taken together, the results highlight the necessity of complete CRS (as opposed to suboptimal debulking) to see the potential benefit of durable locoregional control. It is also important to note that the rate and intent of postoperative systemic therapy was similar between the two groups in our study and is unlikely to be a major factor driving p-PFS results.

Since the presence of EPM significantly reduced OS, we sought to delineate subgroups with survival comparable to that in patients with PDO. On subgroup analysis, patients with peritoneal disease and a single-site EPM (liver and lung) demonstrated OS comparable to those with PDO (median OS 33.7 months vs. not reached and 39.4 months, respectively; p > 0.05). Other studies have demonstrated similar results with respect to OS. Pantelis et al.,^34^ in a large series of patients with stable lung metastases undergoing CRS ± HIPEC, showed that the presence of lung disease did not seem to confer worse outcomes. Similarly, the series by Pawar et al.^13^ found that lung metastases did not influence OS in patients with CRC-PM. Schell et al.^35^ demonstrated that patients with concomitant liver metastases had OS comparable to that of patients with PDO (HR 1.07; 95% CI 0.71–1.62, *p* = 0.733). However, patients with more than two sites of EPM were reported to have a 5-year survival of <15% (vs. 53.3% for PDO). Since 84% of patients in our EPM cohort had single-site metastasis, we could not conduct a further analysis by number of EPM sites.

Adding to the extant literature, our study demonstrates that when complete CRS can be achieved, it provides effective peritoneal disease control on par with that in patients with PDO. In patients with CRC-PM and EPM, peritoneal disease is frequently the determinant of survival and quality of life. Progression of peritoneal disease can cause ascites, abdominal pain, malnutrition, bowel obstruction, need for ostomy or gastrostomy, parenteral nutrition dependence, and ureteral obstruction. Ultimately, peritoneal progression causes limited systemic therapy tolerance and poor candidacy for clinical trials. Locoregional control of peritoneal disease is therefore a priority to allow ongoing opportunities for novel systemic therapies.

Despite the strengths of our analysis, this study has several limitations. This is a single-institution study, so generalizability to other centers in non-academic settings may be limited. A major goal of locoregional therapy for peritoneal disease is preserving or improving quality of life. Yet CRS interventions can be detrimental to quality of life depending on the extent of CRS. We did not analyze quality of life, but it is a major focus of our ongoing work. Retrospective comparative analysis can introduce bias because of measurable and non-measurable confounders. We mitigated the risk of confounding from measured confounders by using entropy balancing. Nonetheless, unmeasured variables can influence patient selection, including physician and disease team experience. We were unable to account for these variables in our analysis. OS analysis is not mature; however, 83% of patients had progression events, allowing meaningful analyses of PFS and p-PFS. Lastly, the small size of the EPM group meant we were unable to delve deeper into prognostic subgroups. Nonetheless, we have presented a meaningful exploratory subgroup analysis that may inform patient selection.

## Conclusion

The findings from the present analysis have important implications for clinical practice. The results support the role of complete CRS in selected patients with limited EPM. Complete CRS is critical to achieve the desired outcomes because the randomized ORCHESTRA trial has already failed to demonstrate the benefit of suboptimal debulking on patient survival or quality of life. We demonstrate here that, if the goal is durable locoregional control, complete CRS can provide success comparable to that seen in patients with PDO. However, it is critical that systemic control of extraperitoneal disease is established before embarking on CRS. Patients should be counseled that, despite aggressive management of EPM, there is a high chance of progression of EPM within 6 months that will require additional systemic therapy or locoregional treatment options. Future studies should evaluate the impact of CRS on quality of life in patients with PDO and EPM. Multi-institutional collaborative and prospective randomized trials are needed to validate these critical findings.
